# Functional methylthio-alkane reductases are widely distributed for bacterial organic sulfur acquisition

**DOI:** 10.1128/mbio.01493-26

**Published:** 2026-10-06

**Authors:** Ana Arroyo Carriedo, Elizabeth A. Morgan, Adrienne B. Narrowe, Rebecca A. Daly, Emily C. Rogers, Sarah J. Young, Kelly S. Bender, Kelly C. Wrighton, Justin A. North

**Affiliations:** 1 Department of Microbiology, Ohio State University 215854, Columbus, Ohio, USA; 2 Department of Crop and Soil Sciences, Colorado State University 124498, Fort Collins, Colorado, USA; 3 School of Biological Sciences, Southern Illinois University 2254, Carbondale, Illinois, USA; University of California Irvine, Irvine, California, USA

**Keywords:** sulfur, nitrogenase, nitrogenase-like enzymes, photosynthetic bacteria, *Clostridium*, anaerobic catabolic pathways, *Fibrobacter*, *Selenomonas*, *Bacillus*, hydrocarbons, methane, ethylene

## Abstract

Methylthio-alkane reductase (MAR) is a relative of nitrogenase that cleaves methylthio-alkanes for methanethiol liberation and subsequent methionine biogenesis. For example, dimethylsulfide, the most abundant volatile organic sulfur compound, is cleaved to methanethiol and methane. To date, the only verified MAR enzymes are from Alphaproteobacteria like *Rhodospirillum rubrum,* despite the long-time observation of gene homologs with potential MAR function present across diverse taxa. Synthesis of MAR genes from uncultivated organisms and expression in *R. rubrum* reveals that functional MAR enzymes are present throughout the Clostridia, Bacilli, Negativicutes, Fibrobacteria, and Bacteroidia classes of bacteria, which range from terrestrial anoxic environments to gut microbiomes. Further, the functional MAR identification screens enabled prediction and validation that *Heliophilum fasciatum* natively possesses a functional MAR, directly demonstrating that MAR enzymes exist in Clostridia. Functional MAR enzymes are only observed in one phylogenetic group, Group IV-C, from the larger nitrogen fixation-like family of sequences, indicating that the remaining phylogenetic groups catalyze alternative and, in many cases, unknown reactions. Finally, either the bona fide NifB of *R. rubrum*, which synthesizes NifB-co, or the homologous MarB associated with each MAR gene cluster can produce an active MAR enzyme, underpinning that nitrogenase-like metallocofactors function at the heart of all MAR systems.

**IMPORTANCE:**

Volatile organic sulfur compounds like dimethylsulfide are ubiquitous and particularly abundant in anoxic terrestrial and gut environments. Further, inorganic sulfur compounds for microbial growth such as sulfate and sulfite can be limited in these environments. Identification of functional methylthio-alkane reductase enzymes from diverse anaerobic bacteria that inhabit anoxic environments reveals that the capacity to utilize volatile organic sulfur compounds and fulfill cellular sulfur requirements is more widespread than previously considered. In addition to catalytic components, MarHDK, either the associated maturase, MarB, or the canonical nitrogenase maturase, NifB, was required, underpinning that *marBHDK* is the minimal gene set for methylthio-alkane reductase across bacteria and that, in general, methylthio-alkane reductases utilize nitrogenase-like cofactors for catalysis.

## INTRODUCTION

**Fig 1:**
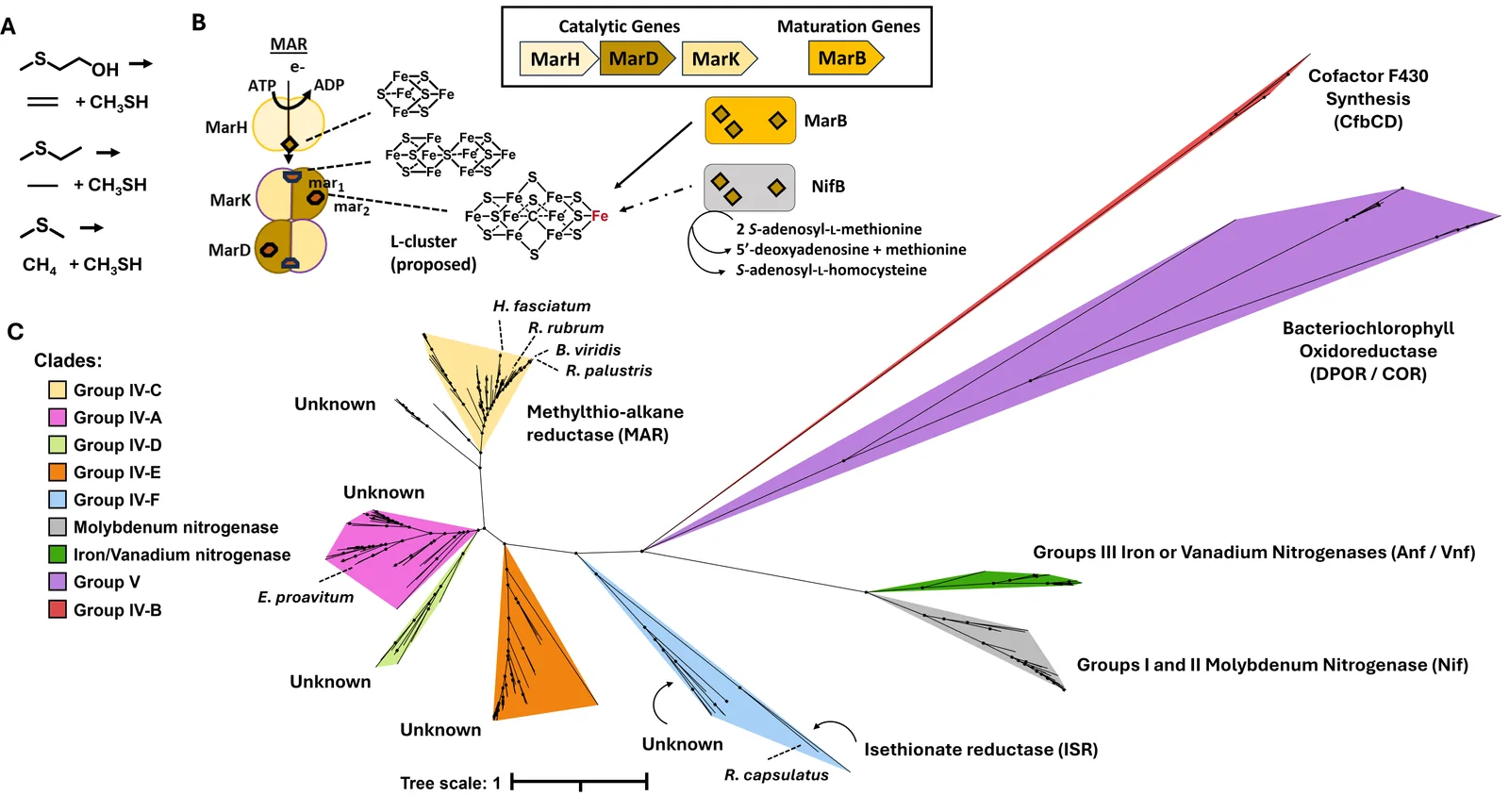
Function of MAR and phylogeny of nitrogenase and nitrogen fixation-like (NFL) sequences. (**A**) Reactions catalyzed by methylthio-alkane reductase (MAR). (**B**) MAR minimal gene set, subunit configuration, and proposed metallocofactors, mar1 and mar2. (**C**) Phylogenetic tree of nitrogenase and NFL sequences based on those identified in reference 1 (File S1).

Methylthio-alkane reductase (MAR) functions by reductively cleaving the carbon-sulfur bond of specific volatile organic sulfur compounds (VOSCs) for microbial sulfur acquisition. Ubiquitous dimethylsulfide (DMS), along with less abundant ethylmethylsulfide (EMS) and (2-methylthio)ethanol (MT-EtOH), can each serve as a substrate, resulting in methanethiol as an immediate precursor to methionine, along with a corresponding hydrocarbon (Fig. 1A) (1, 2). Methane, ethane, and ethylene are each produced stoichiometrically from DMS, EMS, and MT-EtOH, respectively, by MAR. In the phototrophic alphaproteobacterium *Rhodospirillum rubrum*, these gases appear to have no additional function and are released into the environment (1, 3). Other identified bacterial and archaeal enzymes can also cleave DMS for carbon and sulfur metabolism. However, the bacterial DMS monooxygenase, which oxidatively cleaves DMS into methanethiol and formic acid, is explicitly oxygen-dependent (4). Further, DMS methyltransferases that transfer the methyl group of DMS onto coenzyme M as part of methylotrophic methanogenesis are only known in archaea (5). As such, MAR is currently the only known enzyme that can anaerobically cleave the C-S bond of DMS and related VOSCs for bacterial sulfur acquisition in anoxic environments (1, 2). Recently, the MAR system of *R. rubrum* was isolated and characterized (2, 6). Analogous to nitrogenases, MAR is a two-component system composed of a catalytic MarDK heterotetramer (component 1) and a homodimeric ATP-dependent MarH reductase (component 2) (Fig. 1B) (2, 6). The MarDK catalytic core possesses two copies each of complex metallocofactors, mar1 and mar2, that resemble the nitrogenase [8Fe-7S] P-cluster and precursory [8Fe-9S-C] L-cluster (a.k.a. NifB-co), respectively (2, 6). In recent cryo-EM structures, the mar1 cluster in the dithionite-reduced state appeared as a canonical P-cluster with analogous six-cysteine coordination used by nitrogenase (6,6). But in an oxidized state, cluster coordination involved a supernumerary seventh cysteine that is not present in nitrogenase (2). The mar2 cluster electron density envelope and EPR spectra were consistent with an L-cluster, but the presence of a central carbide could not be resolved (6). Given that the precise identities of the MAR cofactors are not fully determined and it is unknown whether they vary among MAR homologs, henceforth we refer to the MAR clusters as the mar1 (P-like) and mar2 (L-like) clusters. For nitrogenase systems, NifB-co specifically refers to the [8Fe-9S-C] L-cluster synthesized by NifB. Based on seminal spectroscopic studies, electrons are passed from the single [4Fe-4S] cluster of the MarH dimer to the mar1 and mar2 clusters for substrate reduction (2). Overall, this process requires ATP, an electron donor (e.g., dithionite as a non-physiological source), and anoxic conditions to prevent catalytic cofactor damage (2, 6). Further, MAR metallocofactor maturation in *R. rubrum* requires either MarB or NifB, the former of which is a NifB homolog (2). Specifically, NifB synthesizes the [8Fe-9S-C] NifB-co through joining two [4Fe-4S] clusters, inserting a central carbide using *S-*adenosyl-L-methionine (SAM), and adding the ninth sulfur (Fig. 1B) (7–9). Given that MarB sequences possess all of the same domains conserved across NifB sequences, it is anticipated that MarB synthesizes an L-like cluster for insertion into MarDK. Lastly, based on genetic evidence, maturation and insertion of the *R. rubrum* MAR metallocofactors by MarB or NifB does not require any intermediate chaperone (Fig. 1B). The nitrogenase cofactor assembly scaffold NifEN, as well as all other potential NifEN homologs in *R. rubrum,* are dispensable for MAR activity. Thus, the minimal MAR system of *R. rubrum* appears to be *marBHDK*, as compared to the *nifBHDKEN* minimal gene set of molybdenum nitrogenase (2, 10, 11).

At present, functional MAR systems have only been verified in the closely related phototrophic purple Alphaproteobacteria *Rhodospirillum rubrum, Rhodopseudomonas palustris,* and *Blastochloris viridis* (1, 3). Given that MAR cannot fix nitrogen and is missing key residues conserved across true nitrogenases for N_2_ substrate coordination, MAR is classified as a nitrogen fixation-like system (NFL) (1, 6, 10). Intriguingly, the NFL family of protein sequences, which contain the MarD sequences as a subset, constitutes a diverse group with distinct phylogenetic clades based on sequence identity of the NflD protein sequences (Fig. 1C) (1, 10, 12). Some of these groups have been well-characterized, including the Group V NFL systems designated as DPOR and COR that catalyze key reduction steps in the synthesis of chlorophyll and bacteriochlorophyll (13, 14). The Group IV NFL family, on the other hand, remains poorly characterized. Due to progressive identification of Group IV members with distinct functions by various researchers in the field, Group IV has been subsequently subdivided over time into lettered clades as reviewed elsewhere (Fig. 1C) (15). Specifically, the Group IV-A NFL system of *Endomicrobium proavitum* is implicated in nitrogen acquisition (16); Group IV-B systems are Ni(II)-sirohydrochlorin-*a,c*-diamide reductive cyclases for synthesis of cofactor F430 in methanogens (17, 18); the aforementioned Alphaproteobacteria MAR systems are located in Group IV-C; and the Group IV-F NFL system of *Rhodobacter capsulatus* is implicated as an isethionate (hydroxyethyl-sulfonate) lyase for sulfur acquisition (19) (Fig. 1C). Notably, the Group IV systems of partial or unknown function exhibit three important features, as observed by earlier bioinformatic genomic analyses. First, is that Group IV NFL genes in general are co-localized with sulfur metabolism genes such as cysteine synthase and methionine synthase, pointing to a possible but unclear role in sulfur metabolism for each of the Group IV clades (1, 10). Second, Group IV NFL genes are distributed across obligate and facultative terrestrial and gut anaerobes, including Clostridia, Negativicutes, Bacilli, Fibrobacter, and Alphaproteobacteria (1, 10). Third, as with nitrogenase genetic systems (*nifBHDKEN*), the Group IV *nflB, nflH, nflD, and nflK* gene homologs are in some cases separated by genes of other function, obfuscating whether *nflBHDK* constitutes the essential functional gene set (1, 10). These observations present the fundamental questions as to whether Group IV-C (Fig. 1C) is the sole MAR-containing clade, whether other Group IV clades also possess functional MAR systems, and whether *marBHDK* appears to be the essential functional gene set across the MAR systems.

Here we report, based on gene synthesis of NFL genes from diverse organisms and heterologous expression in *R. rubrum,* that physiologically relevant levels of MAR activity are observed across Group IV-C sequences. This establishes Group IV-C as the MAR clade of NFL enzymes and indicates that the other Group IV clades, which exhibit low to no MAR activity and in some cases are implicated in other processes (e.g., Group IV-A and IV-F [19, 20]), likely catalyze alternative and yet-to-be-determined reactions. We observe that when the bona fide nitrogenase NifB is absent from *R. rubrum,* heterologous expression of *marB* from the candidate MAR genes is required in addition to *marHDK*. These results reinforce that *marBHDK* is the essential MAR gene set and demonstrate in general that NifB can synthesize a functional metallocofactor that can operate across MAR systems, revealing that cofactors similar to NifB-co function at the heart of MAR systems. Lastly, we observe that NflH homologs form two major clades, one closely related to NifH (Group I NflH) and one more distantly related (Group II NflH), based on sequence identity. Overall, MAR gene clusters with Group I NflH sequences exhibited lower activity when expressed in *R. rubrum* compared to those with Group II NflH sequences, to which the *R. rubrum* MarH belongs. When the candidate heterologous *marB* and *marH* genes were replaced with the native *R. rubrum* homologs, activity increased by orders of magnitude to levels similar to the *R. rubrum* MAR system. This is consistent with the fact that MarB must acquire [4Fe-4S] clusters from cellular iron-sulfur cluster trafficking proteins for metallocofactor synthesis and MarH must acquire electrons from physiological cellular reductant pools (e.g., ferredoxin, flavodoxin), which likely differ between organisms with MAR systems.

## RESULTS

To identify functional MAR systems and microbes that possess them, we took a heterologous expression approach. Nitrogen fixation-like *nflBHDK* gene clusters from organisms that include a Group IV *nflD* gene were synthesized and expressed *in trans* from an expression plasmid in *R. rubrum* as done previously with *R. rubrum* MAR genes (1–3). Specifically, Group IV *nflD* genes and the associated *nflB, nflH*, and *nflK* genes present in organisms’ chromosomal gene neighborhood were selected from across the corresponding Group IV NflD protein family tree (Fig. 1; Groups IV-A, C, D, E, F), except for Group IV-B *cfb* genes for Ni(II)-sirohydrochlorin-*a,c*-diamide reductive cyclase exclusive to methanogens. Selection based on the Group IV NflD family tree as compared to the other NFL subunits was performed given that the NflD sequences are predicted to coordinate the catalytic metallocofactor and primarily confer substrate specificity for a given NFL system. Each *nflBHDK* gene set was designed to be expressed from the constitutive MTA phosphorylase promoter as a polycistronic message always in the order of *nflB, nflH, nflD, and nflK* separated by the same intergenic sequences and translated from the same Shine-Dalgarno sequences (see Materials and Methods; Table S1). The *R. rubrum* deletion strain was used where the native Group IV NFL genes were inactivated from the chromosome to abolish intrinsic MAR activity (1). Also, as necessary, *nifB* was inactivated from the *R. rubrum* chromosome to probe the role of NifB versus other NflB metallocofactor maturases as recently described (2). This approach overcame issues surrounding amplifying and cloning non-contiguous gene sets from genomes of organisms, many of which are uncultured or have no defined media for probing sulfur metabolism from VOSCs in the native host. We considered the same set of NFL gene systems previously identified by bioinformatic analysis of sequenced bacterial genomes (1) (File S1) and tested 40% (20/49) of Group IV-C NFL sequences, as well as 30% (13/43) Group IV-A, 50% (5/9) of Group IV-D, 30% (11/32) of Group IV-E, and 40% (3/8) Group IV-F sequences.

As a benchmark for *in vivo* MAR activity, previously we observed that when DMS, EMS, or MT-EtOH is supplied as the sole sulfur source, the *R. rubrum* or *R. palustris* MAR system expressed from the chromosome or plasmid produced 50–100 µmol of methane, ethane, or ethylene per L culture per culture density (µmol/L/OD) (1, 3). However, not all of the corresponding liberated methanethiol was used by the cells for incorporation into biomass (1, 21, 22). The typical *R. rubrum* cell composition is C_1_:H_1.74_:O_0.42_:N_0.16_:P_0.011_:S_0.001–0.004_ when growing anaerobically and phototrophically in defined minimal media, as also conducted in the present study (23). Therefore, to sufficiently meet cell sulfur needs, one would expect heterologously expressed NFL systems that are viable MAR enzymes to have apparent activities in the range of 40–170 µmol S liberated from VOSCs per gram biomass. Given hydrocarbon production is stoichiometric from VOSCs by MAR and cells range from 0.25 to 0.55 g biomass per L culture per culture density (24, 25), this corresponds to 10–90 µmol hydrocarbon produced by MAR per L culture per culture density (µmol/L/OD). In subsequent heterologous expression and activity screening experiments, 10 µmol/L/OD was used as an approximate theoretical cutoff for identifying a functional MAR system.

### Identification of functional MAR systems across Group IV-C

**Fig 2:**
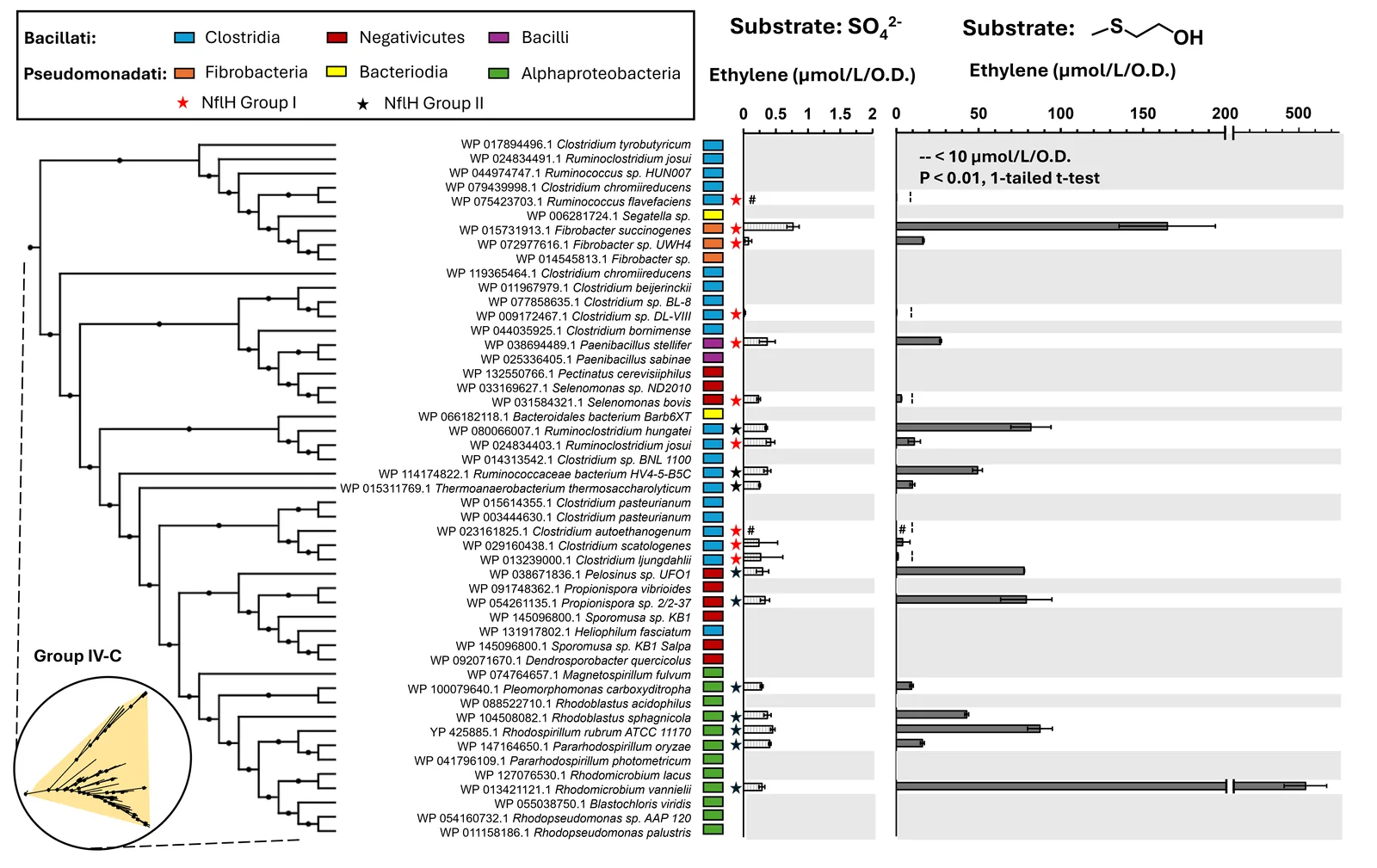
Identification of functional MAR systems within the Group IV-C clade of NflD sequences. The phylogenetic tree is a rectangular representation of the unrooted Group IV-C clade in Fig. 1, with NflD accession numbers and corresponding organisms. Colored blocks represent the taxonomic class for each bacterium. Red and black stars correspond to Group IV-C NflD sequences that are associated with Group I and Group II NflH sequences, respectively, in the NFL gene cluster of the organism (see Fig. S3). Ethylene measurements of *R. rubrum* Δ*mar* Δ*nfl* strain complemented with NFL systems from Group IV-C and grown in the presence of sulfate only (SO_4_^2−^; light gray bars) or sulfate plus (2-methylthio)ethanol (MT-EtOH; dark gray bars). Error bars represent the standard deviation for *n* = 3 biological independent measurements. # indicates no ethylene detected above the 0.01 µmol/L/OD limit of detection. Dashed lines adjacent to ethylene measurement bars indicate those systems with MT-EtOH reduction activities that are statistically lower than the 10 µmol/L/OD cutoff for a physiologically relevant MAR system (*P* < 0.01, one-sample 1-tailed *t*-test).

Previously, identification of functional MAR systems in the closely related *R. rubrum, R. palustris,* and *B. viridis* and the corresponding phylogenetic location of their MarD sequences within the Group IV-C clade led to the proposal that Group IV-C comprises MAR sequences (Fig. 1C) (1, 10). At present, based on recent structural and mutagenesis work of the *R. rubrum* MAR enzyme, only six conserved cysteine residues for mar1 cluster coordination and a conserved cysteine and histidine pair of residues for mar2 cluster coordination are observed to be involved in catalysis (2, 6). These same residues are conserved across nearly all Group IV sequences (1, 2). Thus, specific residues that define MAR substrate specificity from other NFL systems currently of unknown or proposed function are unresolved. Further, phylogenetic distances between potential MAR sequences of the Group IV-C clade strongly correlate with taxonomic classification of the organisms in which they are identified (Fig. 2). This raises the question as to whether more distantly related Group IV-C sequences found across Clostridia, Negativicutes, Bacilli, Fibrobacteria, and Bacteriodota that inhabit anoxic gut and terrestrial environments are functional MAR systems for sulfur acquisition from VOSCs. Therefore, we expressed NFL systems from across Group IV-C in *R. rubrum* growing on sulfate as the sole sulfur source as a control and growing on sulfate plus DMS, EMS, or MT-EtOH to test for MAR activity (Fig. 2; Fig. S1 and S2). Importantly, when MAR genes are expressed *in trans* from the plasmid via the constitutive promoter, the presence of sulfate ensures cell growth but does not repress NFL enzyme activity (1, 2). Further, under these conditions, all *R. rubrum* strains grow to the same final culture density (optical density ~2.0 at 660 nm) in 4 days, and onset of stationary phase at this point arrests NFL activity (1, 2). Subsequent measurement of methane, ethane, and ethylene concentrations produced during growth from the respective VOSC substrate is therefore directly proportional to the MAR activity of the NFL system during growth. It is important to note that this apparent activity is a product of the catalytic rate and protein concentration, the latter of which is unknown given the lack of available specific antibodies or verified non-interfering epitope tags to quantify *in vivo* NFL protein abundance. As such, it is assumed that soluble NFL protein synthesis is uniform across tested systems, but this is not necessarily the case.

Of the 20 NFL systems tested from the Group IV-C clade in the *R. rubrum* NFL gene deletion strain, 15 exhibited MAR activities with MT-EtOH at or above the theoretical 10 µmol per L per culture OD cutoff for a physiologically relevant MAR. Only five were statistically lower than 10 µmol/L/OD (*P* < 0.01, one-sample, 1-tailed *t*-test) (Fig. 2). Similar results were seen for these same systems with DMS and EMS (Fig. S1 and S2), implicating their function as bona fide MAR enzymes. Importantly, high levels of MAR activity (>10 µmol/L/OD) were observed from representatives spanning the Group IV-C clade, revealing that the Group IV-C clade is likely composed entirely of MAR systems. Further MAR systems with high activity were observed for the first time from bacterial members belonging to diverse Clostridia, Bacilli, Negativicutes, and Fibrobacter, along with additional Alphaproteobacteria (Fig. 2). These observations strongly indicate that the anaerobic bacteria possessing the identified functional MAR systems can utilize ubiquitous VOSCs like DMS and MT-EtOH for cellular sulfur demands.

**Fig 3:**
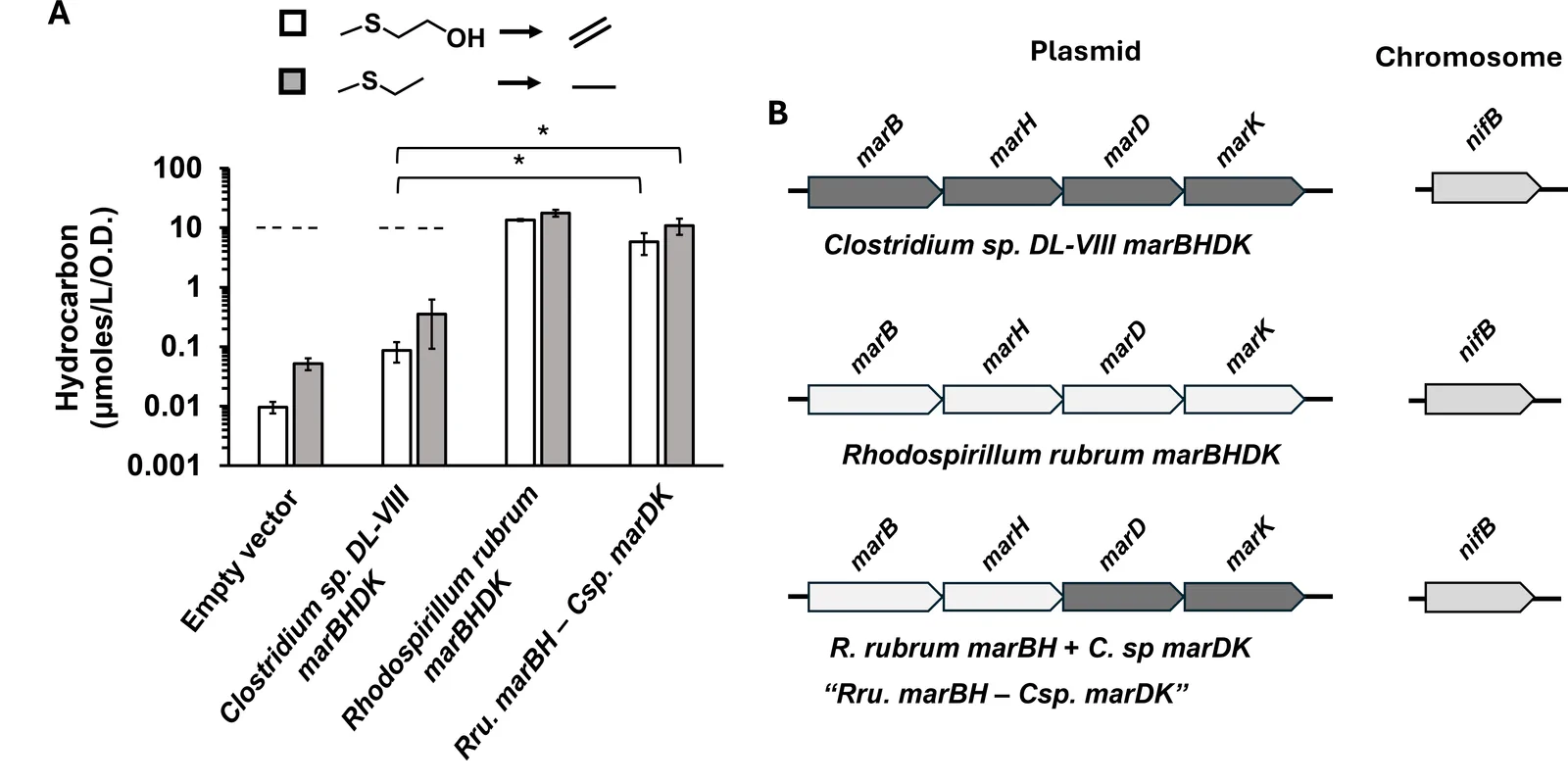
Replacement of *Clostridium* sp. DL-VIII *marBH* with *R. rubrum marBH* results in a functional *Clostridium* sp. DL-VIII MAR system. (**A**) Hydrocarbon measurements of *R. rubrum* Δ*mar* Δ*nfl* strain complemented with empty vector (pMTAP-MCS3), Group IV-C *Clostridium* sp. DL-VIII system plasmid (pMTAP-C4), Group IV-C *R. rubrum* MAR system plasmid (pMTAP-B1), and a chimeric plasmid between *R. rubrum marBH* and *Clostridium* sp. DL-VIII *marDK* (pMTAP-B1C4). Cells were grown in the presence of sulfate and (2-methylthio)ethanol (MT-EtOH) or ethylmethylsulfide (EMS). Error bars represent the standard deviation for *n* = 3 biological independent measurements. (**B**) Gene organization for the plasmids in panel **A**. Dashed lines above hydrocarbon measurement bars indicate MAR activities with the empty vector control or expressed MAR system on the indicated organic sulfur substrate that are statistically below the 10 µmol/L/OD theoretical cutoff for a physiologically relevant MAR system (*P* < 0.01, one-sample 1-tailed *t*-test). * indicates a statistical increase in activity upon replacing *Clostridium* sp*.* CL-VIII *marBH* with *R. rubrum marBH* (*P* < 0.001, 2-tailed *t*-test).

For those Group IV-C NFL systems with low MAR activity (<10 µmol/L/OD), we questioned whether *R. rubrum* could operate these systems, even if properly synthesized. This is because MarB, MarH, and MarDK must be able to acquire [4Fe-4S] clusters from cellular iron-sulfur cluster biosynthetic machinery for catalysis and assembly of the mar1 and mar2 clusters, as observed for nitrogenase (7). Further, MarH must be able to obtain low-potential electrons from the cellular reductant pool, which for *R. rubrum* MarH is implicated to be a ferredoxin (1), but for other MarH homologs may not be the case. Indeed, for nitrogenase, some NifH homologs can support operation of the *Azotobacter vinelandii* molybdenum nitrogenase while others cannot for reasons that are currently not fully resolved (26, 27). Therefore, we initially performed phylogenetic analysis on the NflH sequences to identify any distinct clades that may differ in function. NflH sequences clustered into two distinct major groups: those with closer sequence identity to NifH from *Klebsiella* sp. and *Rhodospirillum* sp., which we designated as NflH Group I, and those with more distant sequence identity, which we designated as NflH Group II (Fig. S3; File S2). Notably, the native MarH of *R. rubrum* is located within NflH Group II. Of the five Group IV-C NFL systems with low MAR activity, all possessed MarH sequences from NflH Group I (Fig. 2), suggesting an incompatibility between NflH Group I proteins and *R. rubrum* as the host. To test this, we replaced the candidate *marB* and Group I *marH* genes of the NFL system from *Clostridium* sp. DL-VIII with *marB* and *marH* of *R. rubrum* (Fig. 3). The original *marBHDK* genes of *Clostridium* sp. DL-VIII expressed in *R. rubrum* exhibited the lowest activities of all the candidate Group IV-C MAR systems tested (i.e., ~0.1 µmol/L/OD ethylene). When the *Clostridium* sp*. marB* and *marH* genes were replaced with those of *R. rubrum*, activity increased nearly 100-fold with MT-EtOH and EMS as substrates to levels that were not statistically less than 10 µmol/L/OD ethylene and ethane (*P* < 0.01; one-sample, 1-tailed *t*-test), demonstrating a functional *Clostridium* sp. MarDK. Importantly, in the *Clostridium* sp*. marBHDK* expression experiments, the *R. rubrum nifB* gene was still present on the chromosome. Given NifB can replace MarB to mature a functional MAR system (2) (see “MarB or nitrogenase NifB are required to mature functional MAR systems from across the MAR family tree” below), an incompatibility of Group I NflH in *R. rubrum* appears responsible for the low activity observed in MAR systems with Group I *nflH* genes. Altogether, these results point to the conclusion that the entire Group IV-C clade comprises functional MAR systems.

### MarB or nitrogenase NifB are required to mature functional MAR systems from across the MAR family tree

**Fig 4:**
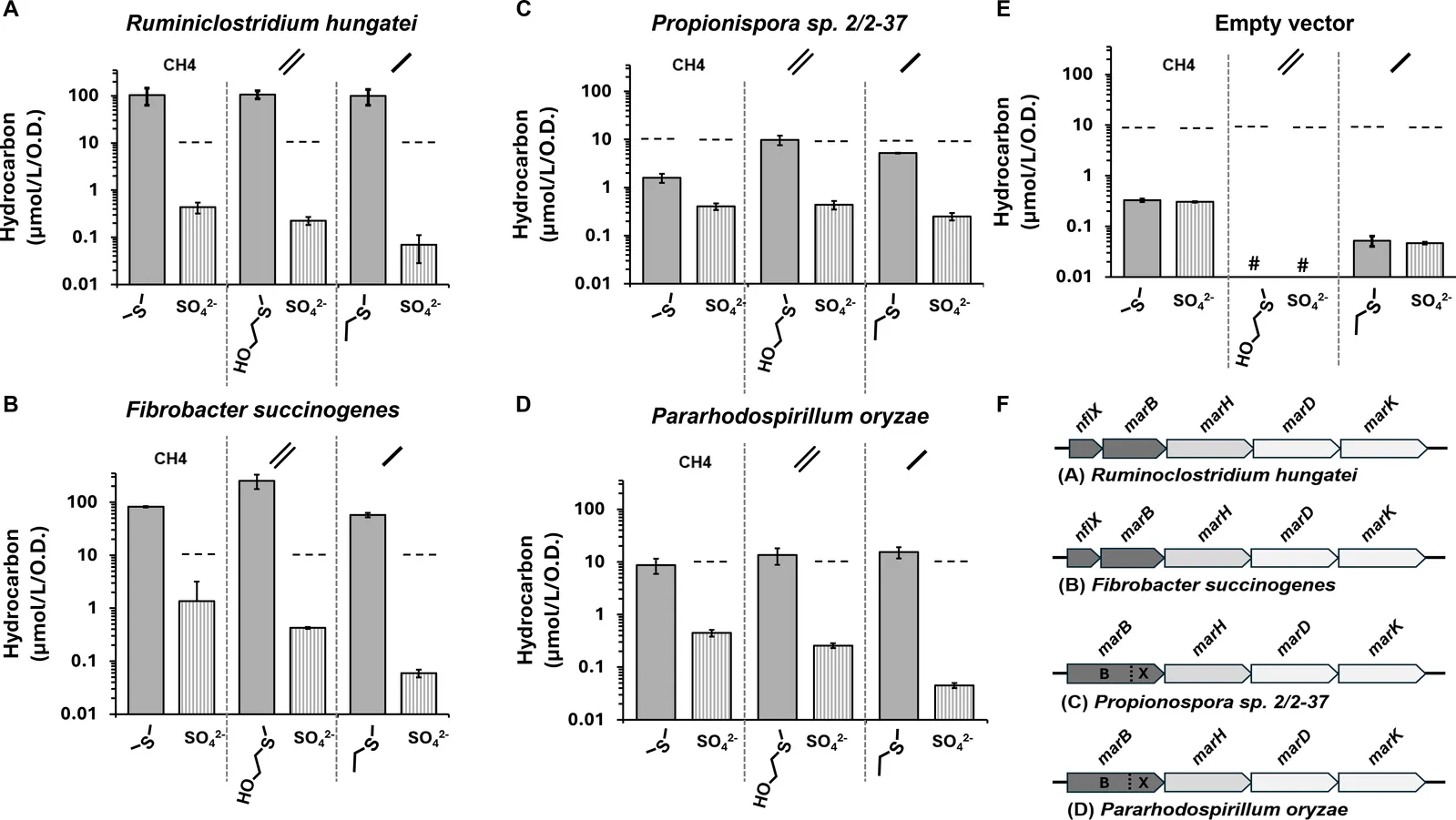
Functional MAR systems with a separate NflX (NifX-like) and truncated MarB protein, or fusion protein of NflX-MarB. (**A–E**) Methane, ethylene, and ethane measurements of *R. rubrum* Δ*mar* Δ*nfl* Δ*nifB* strain complemented with empty vector (**E**) (pMTAP-MCS3), Group IV-C *Ruminiclostridium hungatei* plasmid (**A**) (pMTAP-C5), Group IV-C *Fibrobacter succinogenes* plasmid (**B**) (pMTAP-D6), Group IV-C *Propionispora* sp. plasmid (**C**) (pMTAP-H5), and Group IV-C *Pararhodospirillum oryzae* plasmid (**D**) (pMTAP-A1). Cells were grown in the presence of sulfate or sulfate plus either dimethylsulfide, ethylmethylsulfide, or (2-methylthio)ethanol. Error bars represent the standard deviation for *n* = 3 biological independent measurements. (**F**) Gene organization of the plasmids in panels **A–D**. # indicates no ethylene detected above 0.01 µmol/L/OD limit of detection. -- dashed lines above hydrocarbon measurement bars indicate MAR activities on the sulfate control or indicated organic sulfur substrate that are statistically below the 10 µmol/L/OD theoretical cutoff for a physiologically relevant MAR system (*P* < 0.01, one-sample 1-tailed *t*-test).

In the functional MAR screens (Fig. 2), the NFL deletion strain of *R. rubrum* used retained *nifB* on the chromosome. During the course of these experiments, we discovered and reported that the native MAR system of *R. rubrum* required either MarB or NifB of *R. rubrum* to function and that their role was independent of assembly chaperone NifEN (2). Further, we previously demonstrated that during nitrogenase-repressive conditions (e.g., ammonia), *R. rubrum* still expressed *nifB* and synthesized NifB, resulting in nitrogenase activity that was 100-fold lower than for inducing conditions (e.g., diazotrophic or glutamate growth) (1). Therefore, in the wild-type *R. rubrum* strain, NifB and MarB are present simultaneously. Intriguingly, maturation by NifB shifted *in vivo R. rubrum* MAR specificity toward MT-EtOH reduction. Based on further structural and EPR spectroscopic studies which revealed an L-like mar2 cluster present in MarDK (Fig. 1B) (2, 6), these results suggested that MarB and NifB of *R. rubrum* synthesized either subtly different cofactors or synthesized the same cofactor that was differentially inserted into MarDK in a manner that resulted in altered spheres of coordination (2). This raised the question in our functional screens whether the candidate MarB or native *R. rubrum* NifB was responsible for maturing the observed MAR systems (Fig. 2). Notably, as with true nitrogenases, some MarB sequences are observed to possess a fused NflX (NifX-like) domain, while others are observed to be a truncated MarB, whose gene is often adjacent to a separate *nflX* sequence (Fig. 4F; Table S1) (1, 28, 29). While the precise catalytic function of MarB remains to be determined, based on sequence homology, the radical SAM domain is proposed to be involved in synthesis of an initial [8Fe-8S-C] L*-cluster that may be further converted to a [8Fe-9S-C] L-cluster that ultimately becomes the mar2 cluster, analogous to the formation of NifB-co by NifB (1, 2). The NflX domain fused to MarB or separate NflX protein is anticipated to traffic the mature or intermediate MAR metallocofactor, analogous to nitrogenase cofactor trafficking by NifX (30). Therefore, we determined whether either the MarB-NflX fusion proteins or the separate MarB and NflX proteins were functional in maturing their respective MAR systems when produced in *R. rubrum*.

**Fig 5:**
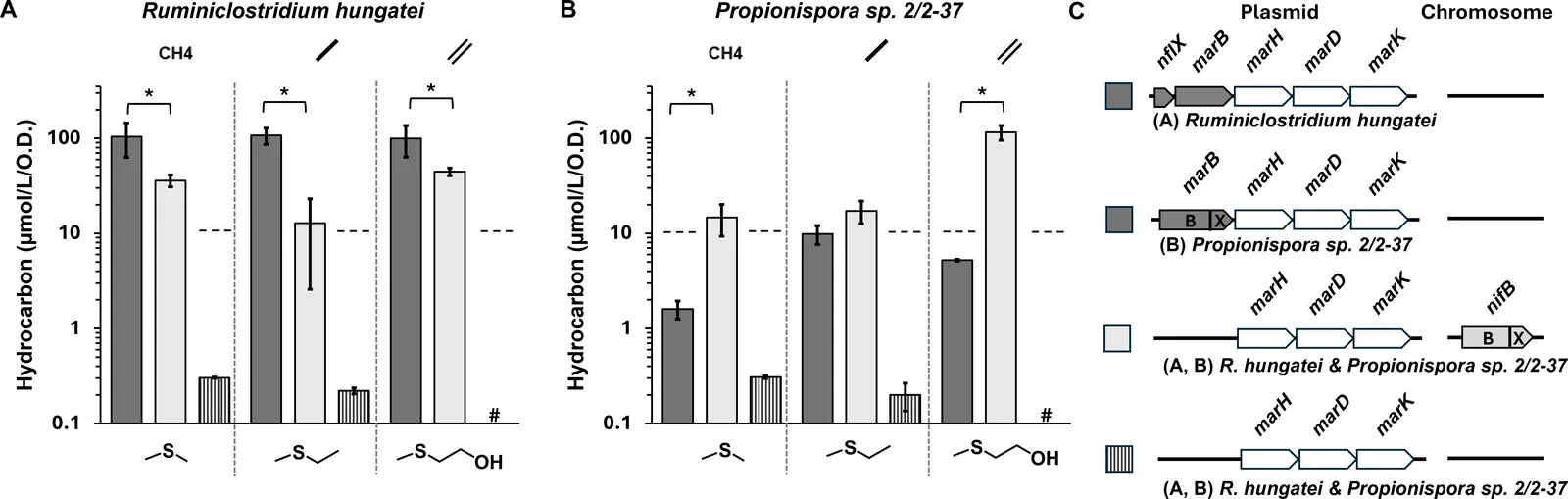
NifB can replace MarB in forming a functional MAR system. (**A and B**) Methane, ethylene, and ethane measurements of *R. rubrum* complemented with the Group IV-C *Ruminiclostridium hungatei* plasmid or the Group IV-C *Propionispora* sp. MAR systems. Dark gray bars indicate that the *marB* gene is present (A, pMTAP-C5; B, pMTAP-H5) and expressed in the *R. rubrum* Δ*mar* Δ*nfl* Δ*nifB* strain, in which *nifB* is absent from the chromosome, as depicted in panel **C**. Light gray bars indicate that the *marB* gene of *R. hungatei* or *Propionispora* sp. is absent from the plasmid (A, pMTAP-C5-marHDK; B, pMTAP-H5-marHDK) and is expressed in *R. rubrum* Δ*mar* Δ*nfl* strain, in which *nifB* is present on the chromosome, as depicted in panel **C**. Hashed bars are the same *marHDK* expression plasmids (A, pMTAP-C5-marHDK; B, pMTAP-H5-marHDK) expressed in the *R. rubrum* Δ*mar* Δ*nfl* Δ*nifB* strain, in which neither *marB* nor *nifB* is present. Cells were grown in the presence of sulfate and either dimethylsulfide, ethylmethylsulfide, or (2-methylthio)ethanol. Error bars represent the standard deviation for *n* = 3 biological independent measurements. (**C**) Plasmid and chromosome gene organization for the plasmids in panels **A and B**. # indicates no ethylene detected above 0.01 µmol/L/OD limit of detection. Dashed lines above hydrocarbon measurement bars indicate MAR activities on the indicated organic sulfur substrate that are statistically below the 10 µmol/L/OD theoretical cutoff for a physiologically relevant MAR system (*P* < 0.01, one-sample 1-tailed *t*-test). * indicates MAR activities that statistically change upon maturation with MarB versus NifB (*P* < 0.001, 2-tailed *t*-test).

To test MarB functionality from MAR systems across the Group IV-C family tree, we selected one representative MAR system from each of the Fibrobacter, Clostridia, Negativicutes, and Alphaproteobacteria classes of bacteria, which spanned the microbial diversity and Group IV-C phylogenetic tree distances of MAR systems (Fig. 2 and 4). These gene clusters included either the *marBHDK* paradigm, where *marB* possesses a fused *nflX* sequence, or the *nflx*/*marBHDK* paradigm, where *marB* is truncated and a *nifX-*like gene is separate (*nflX*; Fig. 4F). The MAR genes were then expressed in the *R. rubrum* NFL and *nifB* deletion strains (Fig. 4). The MAR systems of *Ruminoclostridium hungatei* and *Fibrobacter succinogenes,* which possess separate *marB* and *nflX* genes, were functional in *R. rubrum* with similar activities regardless of whether NifB was present or absent. Similarly, the MAR systems of *Propionispora* sp. and *Pararhodospirillum oryzae*, which alternatively use the MarB-NflX fusion, were likewise functional in the presence or absence of NifB. Further, when the *marB* genes of *R. hungatei* and *Propionispora* sp. were omitted, *marHDK* genes expressed in the *R. rubrum* NFL and *nifB* deletion strain showed no MAR activity beyond empty vector controls (*P* > 0.1; 2-tailed *t*-test) (Fig. 4E and 5). Taken together, these results demonstrate that versions of MarB with and without a fused NifX-like domain are functional for maturing MAR, presumably by synthesizing the mar2 catalytic metallocofactor. However, precisely how the fused or separate NifX-like protein functions in MAR metallocofactor trafficking and whether the native nitrogenase NifX protein of *R. rubrum* (Rru_A2284) can also perform the same function is unknown.

With the MarB homologs from across the Group IV-C clade of MAR systems established as necessary components for MAR activity in the absence of NifB, we subsequently were positioned to determine whether NifB instead could form a functional MAR system. We introduced into the *R. rubrum* NFL deletion strain the plasmids for the *R. hungatei* and *Propionispora* sp. MAR systems in which the *marB* and *nflX* genes were removed (Fig. 5). With *marB* removed, only *nifB* present on the chromosome was available to mature a functional MAR system. When NifB was present, MAR activity by the expressed *R. hungatei* or *Propionispora* sp*. marHDK* was greater than 10 µmol/L/OD, indicating that NifB in general can mature a functional MAR system (Fig. 5). Further, for the *Propionispora* sp. system, MAR activity increased by 10-fold for DMS and MT-EtOH in the presence of NifB versus MarB (*P* < 0.001, 2-tailed *t*-test), while activity with EMS remained the same (MAR matured by MarB and EMS as substrate = 10 ± 3 µmol ethane/L/OD, MAR matured by NifB and EMS as substrate = 17 ± 5; *P* > 0.1, 2-tailed *t*-test). In contrast, MAR activity of the *R. hungatei* system decreased for all substrates when NifB versus MarB was present (*P* < 0.001, 2-tailed *t*-test). Previously, we observed that the *R. rubrum* native MAR system synthesized in the presence of NifB versus MarB showed increased activity with MT-EtOH and decreased activity with DMS (2). Thus, NifB clearly can mature functional MAR enzymes across the MAR family of systems, but with differences in activity profiles. This underpins a common functional link between NifB and MarB proteins but also suggests possible differences in maturase activity or cofactor trafficking and insertion that remain to be elucidated.

### Methylthio-alkane reductase activity appears localized to the Group IV-C MAR systems

**Fig 6:**
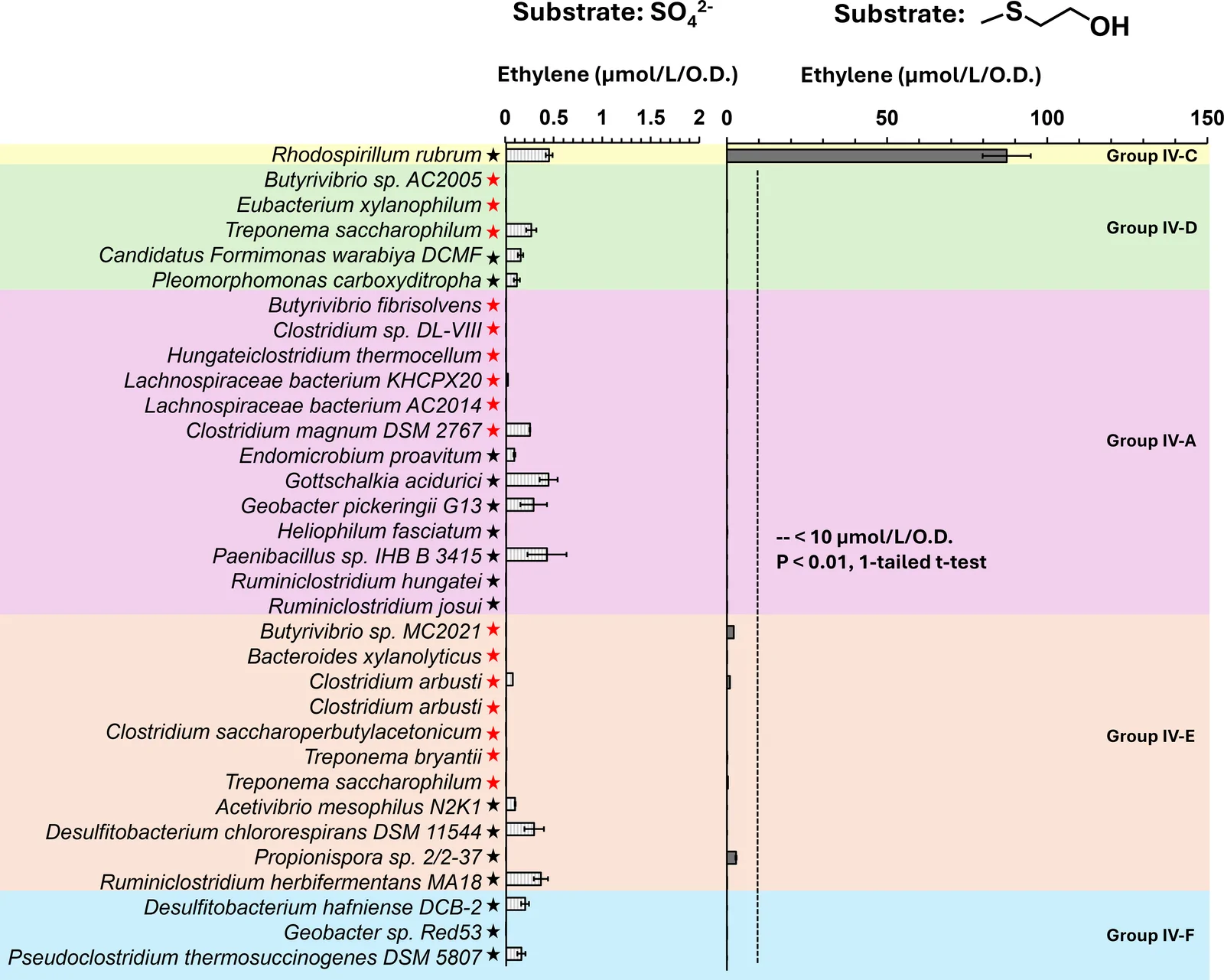
MAR activity is not observed in NFL systems from phylogenetic clades beyond Group IV-C. Colored blocks represent the NFL clades as color-coded in Fig. 1. Red and black stars correspond to Group IV NflD sequences that are associated with Group I and Group II NflH sequences, respectively, in the NFL gene cluster of the organism (see Fig. S3). Ethylene measurements of *R. rubrum* Δ*mar* Δ*nfl* strain complemented with NFL systems from Group IV-A, IV-D, IV-E, and IV-F grown in the presence of sulfate only (SO_4_^2−^; light gray) or sulfate plus (2-methylthio)ethanol (dark gray). Error bars represent the standard deviation for *n* = 3 biological independent measurements.Dashed lines adjacent to ethylene measurement bars indicate those systems with MT-EtOH reduction activities that are statistically lower than the 10 µmol/L/OD cutoff for a physiologically relevant MAR system (*P* < 0.01, one-sample 1-tailed *t*-test).

With the identification of Group IV-C NFL sequences as a MAR clade, we questioned whether other groups within the nitrogen fixation-like family of sequences also exhibited MAR activity (Fig. 1). For example, Groups I, II, and III are all composed of the true molybdenum, vanadium, and iron nitrogenases (Fig. 1). Thus, it is possible that multiple Group IV NFL clades may likewise be composed of MAR systems. Using the same approach as for the Group IV-C NFL sequences, we tested *nflBHDK* gene clusters for NFL systems from the Group IV-A, IV-D, IV-E, and IV-F clades, primarily of unknown function (Fig. 6; Fig. S4 and S5). As previously discussed, *nfl* genes are found across a wide range of bacteria with different living environments, ranging from soil microbes like *R. rubrum* to gut microbes like *Endomicrobium proavitum*. Therefore, the screen included sequences from a wide range of bacterial classes, including Alphaproteobacteria, Negativicutes, Fibrobacteria, Bacilli, Clostridia, and Bacteroidia. Further, these candidate gene clusters contained NflH members from both the NflH Group I and NflH Group II clades. All of the candidate NFL gene clusters were expressed *in trans* from the same plasmid system as the Group IV-C MAR genes in the *R. rubrum* NFL gene deletion strain. In every case across the Group IV-A, IV-D, IV-E, and IV-F clades, ethylene production activity from MT-EtOH and ethane production activity from EMS were statistically less than 10 µmol/L/OD (*P* < 0.01, 1-tailed *t*-test). Similarly, methane production activity from DMS was statistically less than 10 µmol/L/OD in all cases save for Group IV-A *Clostridium magnum*. Further, nitrogenase shows no measurable activity with DMS, EMS, and MT-EtOH; therefore, these low activities cannot be from the native nitrogenase (6). These low MAR activities observed from Group IV-A, IV-D, IV-E, and IV-F systems could be due to several factors. Group IV-C is likely the primary MAR clade, but the remaining clades of unknown or proposed function remain to be elucidated.

### *Heliophilum fasciatum*, a phototrophic Clostridia, possesses a functional MAR

**Fig 7:**
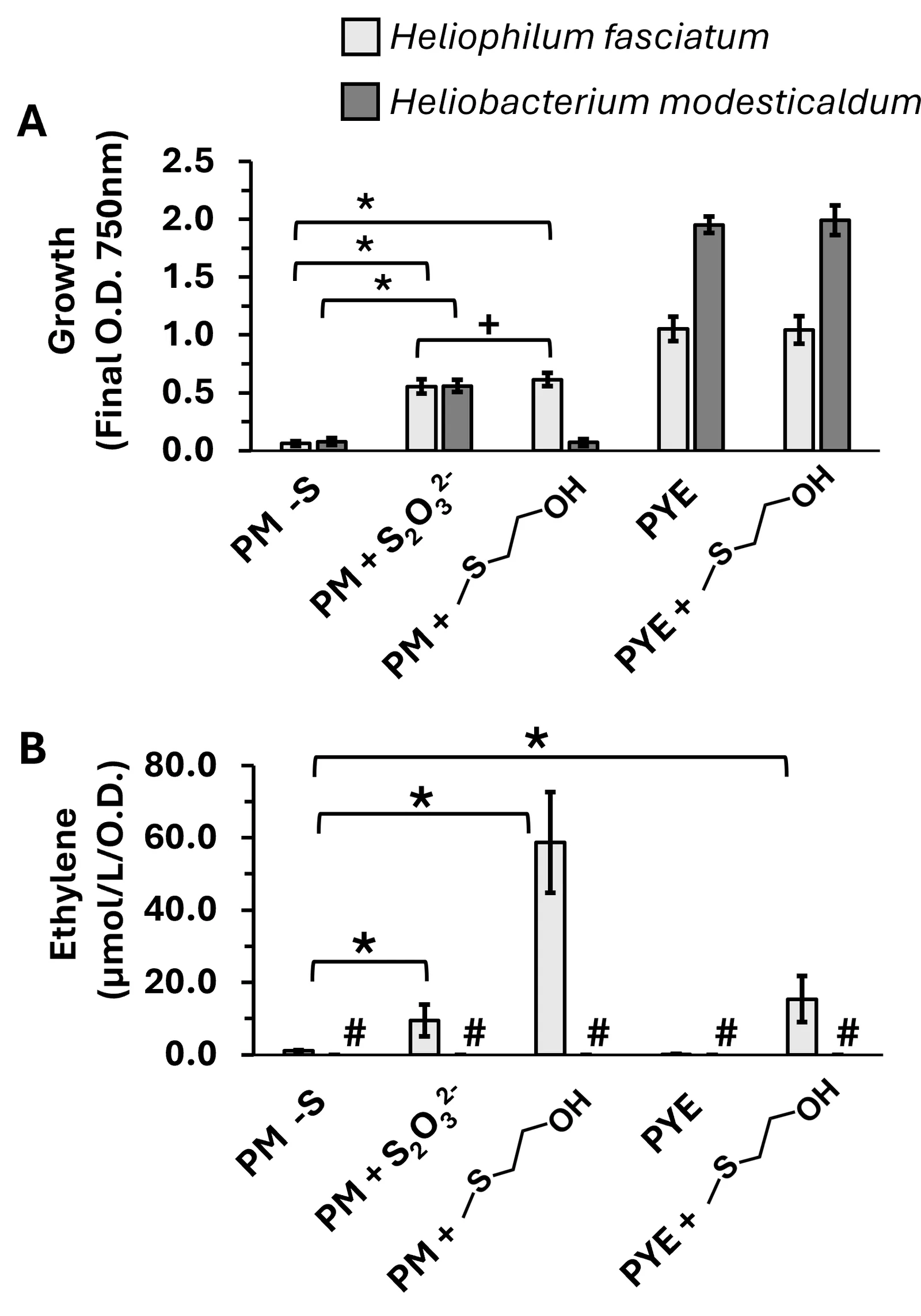
The Clostridia, *Heliophilum fasciatum* natively possesses a MAR system. (**A and B**) Final optical density and ethylene produced by *Heliophilum fasciatum* and *Heliobacterium modesticaldum* when cultured in pyruvate-defined medium (PM) or rich peptone-yeast extract media (PYE) supplemented with thiosulfate or (2-methylthio)ethanol as the sole sulfur source in the case of PM or an additional sulfur source in the case of PYE media. # indicate no ethylene detected above 0.01 µmol/L/OD. Error bars represent the standard deviation for *n* = 3 biological independent measurements. * indicates a statistically significant increase in (**A**) growth on PM or (**B**) MAR activity relative to no-sulfate (PM-S) control (*P* < 0.01, 2-tailed *t*-test). + indicates no statistically significant difference (*P* > 0.1, 2-tailed *t*-test).

Heliobacteria are obligate anaerobes and form a unique order, Heliobacteriales, within Clostridia. Heliobacteria are unusual gram-positive phototrophs that are a distinct lineage from the purple and green phototrophic proteobacteria. They lack any known autotrophic pathways for CO_2_ fixation but rather exhibit heterotrophic and often extremophilic lifestyles with environmental pH ranging from 6.5 to 9 and temperatures ranging from 30–50°C (31). *Heliobacterium modesticaldum* from volcanic soils and hot springs possesses a molybdenum nitrogenase (Nif) system but no Group IV NFL systems (32, 33). Heliobacteria possessing nitrogenase are capable of diazotrophic growth (34, 35). In contrast, *Heliophilum fasciatum* from rice paddies and soils does not possess nitrogenase but rather possesses multiple Group IV NFL systems, including a system with candidate Group IV-C MarD as associated Group II NflH (31, 35). While genetic tools are not available, developed defined media with pyruvate as the carbon source enable controlled experiments to measure MAR activity with VOSCs directly for the first time in a *Clostridia* species (35). We grew *H. modesticaldum* and *H. fasciatum* in rich media with and without MT-EtOH and in defined media with thiosulfate or MT-EtOH as the sole sulfur source (Fig. 7). Given that these organisms form strands and bundles during growth, acquisition of conventional growth curves was not feasible. Therefore, as done previously, cultures were allowed to grow to completion and then vigorously vortexed to disperse the cells for a final culture density measurement (35). The MAR-lacking *H. modesticaldum* was incapable of growth with MT-EtOH as the sole sulfur source, and in all conditions tested, no ethylene or any other hydrocarbon was observed (Fig. 7). In stark contrast, *H. fasciatum* grew to the same final density with MT-EtOH or thiosulfate as the sole sulfur source. Concomitantly, *H. fasciatum* cells grown on MT-EtOH exhibited MAR activities for ethylene production of 60 µmol/L/OD, as one would expect for a functional MAR system that fulfills the sulfur needs of the cell. Further, growth on sulfate as the sole sulfur source or growth in rich media supplemented with MT-EtOH exhibited suppressed MAR activities, as observed with *R. rubrum* (1), consistent with cellular sulfur needs being preferentially fulfilled by inorganic sulfur sources when present. Altogether, these results directly demonstrate MAR activity in a Clostridia species with a Group IV-C MAR system, fully corroborating the functional MAR screens in *R. rubrum* (Fig. 2).

## DISCUSSION

Previously, methylthio-alkane reductase activity was only known to occur in select anoxic phototrophic Alphaproteobacteria common to freshwater and terrestrial soils (1). The functional MAR screens of candidate genes heterologously produced in *R. rubrum* and the direct quantification of MAR activity in the Clostridia, *Heliophilum fasciatum*, reveal that functional MAR systems are spread across diverse bacteria (Fig. 2 and 7). Importantly, MAR systems appear to be localized to the Group IV-C clade of the NFL family of sequences (Fig. 5). While some candidate MAR sequences, particularly those with NflH Group I sequences for the MarH reductase component, exhibited lower-than-expected activities for a MAR system (<10 µmol/L/OD), replacement of the *Clostridium* sp. DL-VIII MarB and MarH with that of *R. rubrum* resulted in expected MAR activities. By extension, those other Group IV-C NFL systems with low observed MAR activity, particularly those with an NflH Group I, are highly likely to be functional MAR systems as well, further supporting the conclusion that the entire Group IV-C clade comprises MAR systems (Fig. 3). At present, the precise details as to why MarH and/or MarB of *R. rubrum* can support MAR activity of the *Clostridium* sp. DL-VIII MarDK while the original *Clostridium* MarB and/or MarH cannot when heterologously produced in *R. rubrum* is unknown.

Initial work revealed that the *R. rubrum* MarB or NifB could mature the native *R. rubrum* MAR system into a functional enzyme (2). In the present study, we further observed that the NifB of *R. rubrum* can function in lieu of MarB for distantly related MAR systems from Clostridia and Negativicutes. Given that NifB synthesizes the [8Fe-9S-C] NifB-co, these results further underpin the conclusion that a nitrogenase-like cluster is the catalytic metallocofactor employed across the MAR family of enzymes. Such observations are also consistent with the fact that *R. rubrum* MarD Cys270 and His429 residues are required for the mar2 cluster coordination (2, 6), and are conserved across all Group IV-C MarD sequences (1). Differences in metallocofactor identity or protein coordination sphere effects on MAR substrate activity profiles when MAR is assembled with NifB versus MarB remain to be determined (Fig. 5). Regardless, it appears that MarB is present across all bioinformatically identified MAR systems to assemble a MAR with specific VOSC substrate activities (Table S1) (1).

The lack of any MAR activity by NFL systems outside of Group IV-C has two possible interpretations. It may be that *R. rubrum* is incapable of synthesizing a functional enzyme from these NFL gene systems that are more distantly related to the *R. rubrum* MAR as compared to the more closely related Group IV-C MAR sequences. At present, this is impossible to rule out. However, the more probable explanation is that these Group IV-A, IV-D, IV-E, and IV-F sequences are not methylthio-alkane reductases and rather catalyze alternative biological reactions. This alternative function model is consistent with the observation that Group IV-A contains a sequence from *Endomicrobium proavitum* implicated in nitrogen fixation (16), and Group IV-F possesses a sequence from *Rhodobacter capsulatus* involved in cleavage of isethionate (hydroxyethane sulfonate) for bacterial sulfur acquisition (19) (Fig. 1). Even *R. rubrum* possesses both the Group IV-C MAR system and a Group IV-A NFL system, the latter of which has no observed MAR activity (1). Given that nearly all of the Group IV-A, IV-D, IV-E, and IV-F NFL systems are also co-localized with sulfur metabolism genes, they are still implicated in sulfur metabolism, albeit of unknown function.

Dimethylsulfide (DMS), the most abundant VOSC, is commonly found in anoxic environments. In the rat gut, DMS is present at a concentration of ~10 nM (36). In the rumen of cattle, DMS concentrations range from 1–100 µM depending on feed type (37, 38), and in anoxic sediments and bog waters, DMS concentrations range from 1–300 nM (39–41). *Fibrobacter* sp*.*, *Selenomonas* and *Propionispora* spp. (Negativicutes), and *Ruminiclostridium* sp. (Clostridia) with functional MAR enzymes identified in this study are commonly found in gut and rumen microbiomes. Similarly, Alphaproteobacteria like *Rhodoblastus sphagnicola* and *Pararhodospirillum oryzae*, Negativicutes like *Pelosinus* sp., Bacilli like *Paenibacillus stellifer*, and Clostridia like *Heliophilum* sp. are common inhabitants of anoxic soils and groundwaters. Interestingly, while DMS is also a major component of the oceanic sulfur cycle (42–44), no MAR or any other Group IV NFL systems have been observed in marine bacteria (1). However, oceanic sulfate is at sufficient levels for microbial growth (28 mM), as reported in reference 44, whereas inorganic sulfur like sulfate and sulfite can be limited (<100 µM) in anoxic freshwater and waterlogged soil environments (reviewed in reference 3). Lastly, as seen for both *R. rubrum* (1) and *H. fasciatum*, MAR activity is repressed when alternative sulfur sources are available like sulfate, or cysteine and methionine (provided as peptone, PYE; Fig. 7). Therefore, altogether these observations point to a model where diverse anaerobic organisms with MAR turn to VOSCs like DMS and MT-EtOH in their anoxic water, soil, and gut environments when preferred organic and inorganic sulfur sources become limiting.

## MATERIALS AND METHODS

### Chemicals and helper enzymes

All chemicals were of reagent purity and were obtained from Sigma-Aldrich, except for ethylmethyl sulfide, which was from Tokyo Chemical Industries, and 2-(methylthio)ethanol, which was from Thermo Fisher. All restriction enzymes, T4 ligase, and Gibson Assembly Kit were from New England Biolabs, and Phusion DNA polymerase was from Thermo Fisher.

### Bacterial strains and growth conditions

All *Rhodospirillum rubrum* strains used in this study are derived from wild-type strain S1 (ATCC 11170) as follows: ΔmarBHDK ΔnflDK (genotype: ΔRru_A0793:Rru_A0796, ΔRru_A0772:Rru_A0773, StR [1]); ΔmarBHDK ΔnflDK ΔnifB (genotype: ΔRru_A0793:Rru_A0796, ΔRru_A0772:Rru_A0773; ΔRru_A0994; StR [2]).

*Heliophilum fasciatum* and *Heliobacterium modesticaldum* were the wild-type strains, as previously reported (32, 35).

*Rhodospirillum rubrum* strains, each with a pMTAP-based plasmid, were initially grown aerobically in Ormerod’s malate minimal medium supplemented with 0.3% peptone and yeast extract (45). All *R. rubrum* strains were transformed with each plasmid by conjugative mating exactly as previously described (3). For growth and quantification of MAR activity, transformed *R. rubrum* strains were grown anaerobically in Hungate tubes with appropriate antibiotics (50 µg/mL streptomycin and 2 µg/mL tetracycline). Cultures were then washed and transferred to anaerobic culture tubes with a 95:5 N_2_:H_2_ gaseous headspace at a starting density of 0.05 (OD at 660 nm) in Ormerod’s sulfur-free malate minimal medium supplemented with 1 mM (NH_4_)_2_SO_4_ and/or 1 mM VOSC. Ormerod’s medium was made sulfur-free by replacing all sulfate-complexed metals with chloride versions (45). All strain manipulations were performed in a Coy anaerobic chamber with a 95:5 N_2_:H_2_ atmosphere. All strains were grown at 30°C under 2,000 lux for growth and MAR activity experiments. Growth was monitored by optical density at 660 nm until stationary phase was achieved. For all strains, this occurred within 96 h, with growth to a final OD at 660 nm of ~2.0.

*Heliophilum fasciatum* and *Heliobacterium modesticaldum* cultures were grown in peptone-yeast extract (35) or defined minimal pyruvate medium consisting of 20 mM sodium pyruvate, 0.034 mM EDTA-disodium salt, 0.36 mM MgCl_2_·2H_2_O, 0.5 mM CaCl_2_·2H_2_O, 20 mM NH_4_Cl, 5.2 mM K_2_HPO_4_, 4.4 mM KH_2_PO_4_, 20 µM chelated FeCl_3_·6H_2_O solution, 15 µg/L biotin, 20 µg/L vitamin B12, 12.5 nM Na_2_SeO_3_, 50 µg/L para(4)-benzoic acid, and 1 mL/L trace elements (per L: 10 g EDTA-disodium salt, 0.8 g MnCl_2_·4 H_2_O, 0.4 g H_3_BO_3_, 0.4 g NaMoO_4_·2H_2_O, 0.2 g ZnCl_2_, 0.2 g NiSO_4_·6H_2_O, 0.08 g CoCl_2_·6H_2_O, 0.02 g CuCl_2_·2H_2_O, 0.02 g VOSO_4_·2H_2_O, 0.02 g Na_2_WO_4_·2H_2_O). Prior to autoclaving, the medium was boiled for 30 min under nitrogen before being transferred to anaerobic culture tubes, followed by stoppering and metal crimping. Individual sulfur sources, where indicated, were added separately to tubes of medium from anaerobic stocks to a final concentration of 1 mM for thiosulfate and MT-EtOH. An inoculum volume of 0.2 mL from active cultures grown on the same sulfur source for at least two transfers was added to 9.8 mL of anaerobic medium and incubated at 32°C with incandescent illumination until stationary phase was reached, as monitored by OD measurements at 660 nm. At this point, cells were thoroughly vortexed to disrupt bundles and fibers into individually dispersed cells for final OD measurement at 750 nm to avoid spectroscopic overlap with bacteriochlorophyll *g* of *Heliobacteria* sp. (32, 35).

### Plasmid construction for expression of NFL gene clusters

All NFL genes and protein accessions corresponding to sequence homologs of *nifX, nifB, nifH, nifD,* and *nifK* are provided in Table S1. Each of these genes was previously identified in reference 1. Briefly, the *R. rubrum* MarB, MarH, MarD, and MarK proteins were separately queried against the NCBI reference genome database using the translated nucleotide BLAST (tblastn) algorithm to retrieve NflB, NflH, NflD, and NflK sequences. Each identified NflB, NflH, NflD, and NflK candidate was correlated with its reference genome to identify gene clusters with all four candidate genes, and any *nflX* (*nifX*-like) sequences associated with *nflB* sequences were also retained. Each gene sequence was modified to remove any NdeI, XhoI, and KpnI sequences while retaining codon identities. Then each gene sequence was codon optimized using the *R. rubrum* codon frequency table and a balanced optimization strategy that incorporates *R. rubrum* codons into the refactored genes at the same rate observed in *R. rubrum* using the JGI Boost 2.0 software (46). Gene sequences were always arranged in the order of *nflX* (if applicable), *nflB, nflH, nflD, nflK*, with identical intergenic regions between each gene, resulting in ~5,000 bp gene cluster sequences. Specifically, when *nflX* was present, the 5′-UTR of *R. rubrum marB* was used between *nflX* and *nflB*: 5′-GTTTCCGCCA**AGGAGA**CGGCTTTC (Shine-Dalgarno in bold). Between *nflB* and *nflH,* the *R. rubrum marB* and *marH* intergenic region was used: 5’- AGCCCGTGTC**AGGACG**AGAGAACC. For *R. rubrum,* the native intergenic regions between *marH*, *marD*, and *marK* are only 8–12 bp, with the Shine-Dalgarno sequence overlapping the nonsense codon of the upstream gene. Therefore, between *nflH* and *nflD,* the Rru_A0360 5′-UTR was used: GGTTTTTACAAGGATGATggtacc, and between *nflD* and *nflK,* the *R. rubrum* Rru_A0792 5′-UTR was used: CCGTCGCCTCA**AGGAGG**GACAGAGG. The Rru_A0360 (*mtnA*) and Rru_A0792 (*oahs*) 5′-UTRs were chosen due to these genes’ known roles in and expression during MT-EtOH metabolism, as with the MAR genes (1, 3). Finally, the intergenic region between *nflH* and *nflD* contained a KpnI site (ggtacc) for sub-cloning of *marBH* and *marDK* fragments. The entire gene clusters were synthesized by Twist Biosciences and cloned into plasmid pMTAP-MCS3 (3) by Gibson Assembly. The Gibson Assembly incorporated the start codon of the first gene of the gene cluster into the NdeI site of the Rru_A0361 (*mtnP*) promoter in pMTAP-MCS3 and incorporated the 3′-UTR of the last gene into the XhoI site of pMTAP-MCS3. Plasmids were verified by Sanger sequencing and stored in *E. coli* Stellar cells. Plasmid names and full codon-optimized sequences are provided in Table S1.

### Construction of chimeric *R. rubrum* marBH and *Clostridium* sp. marDK expression plasmid

Plasmid pMTAP-C4 with Group IV-C *marBHDK* homologs from *Clostridium* sp. DL-VIII and plasmid pMTAP-B1 with *R. rubrum* ATCC 11170 *marBHDK* were digested with

NdeI and KpnI, and the pMTAP-C4 digestion reaction was also treated with Antarctic phosphatase (New England Biolabs). The *marBH* fragment of *R. rubrum* and the vector backbone of pMTAP-C4 containing only the *Clostridium* sp*. marDK* were isolated by agarose gel purification and QIAquick Gel Extraction Kit (Qiagen). Fragments were ligated together using T4 ligase to form plasmid pMTAP-B1C4 (Table S2) and transformed into *E. coli* Top10 cells (Invitrogen). Transformants were selected on LB agar plates with 8 μg/mL tetracycline, and recovered plasmids were verified via Sanger sequencing.

### Construction of marHDK expression plasmids

*Ruminiclostridium hungatei* plasmid pMTAP-C5 and *Propionispora* sp. 2/2-37 plasmid pMTAP-H5 were used as templates to amplify only their *marHDK* genes by PCR using the primers listed in Table S3. The PCR fragments and plasmid pMTAP-MCS3 were digested with NdeI and KpnI, and plasmid pMTAP-MCS3 was also treated with Antarctic phosphatase. Each *marHDK* fragment was ligated into digested pMTAP-MCS3 using T4 ligase to construct plasmids pMTAP-C5-marHDK and pMTAP-H5-marHDK expressing the *R. hungatei* and *Propionispora* sp*. marHDK* genes, respectively (Table S2). Plasmids were transformed into *E. coli* Top10 cells and verified by Sanger sequencing as described above.

### Gas chromatography analysis of hydrocarbons

Quantification of methane, ethane, and ethylene was performed using a Shimadzu GC-14A with Restek Rt-Alumina BOND/Na_2_SO_4_ column, 30 m, 0.53 mm inner diameter. Gaseous culture headspace after growth experiments was injected at 180°C and separated isothermally at 35°C. Eluted compounds were detected by a flame ionization detector at 180°C and identified on the basis of the retention times of methane, ethane, and ethylene standards (Schott; Air Liquide). The total amount of each hydrocarbon present was calculated from the peak area as compared with standard concentration curves made from the corresponding Schott reference standard.

### Phylogenetic analysis

For phylogenetic analysis of NflD and NflH sequences and phylogenetic tree construction, all NflD and NifD or all NflH and NifH sequences were aligned by Clustal Omega (47, 48). Phylogenetic trees were then constructed using MEGA11 (v. 11.0.13) with an LG + F model of evolution, a G + I site substitution rate, and 100 bootstrap iterations (49). NflD and NflH family trees with NCBI sequence accession numbers were output in Newick format (Files S1 and S2) and visualized in iTOL (v. 7.6) (50).

## Data Availability

All strains and plasmids will be made available upon request

## SUPPLEMENTAL MATERIAL

File S1 mbio.01493-26-s0001.txt Phylogenetic tree of NifD and NflD sequences and their accession numbers.

File S2 mbio.01493-26-s0002.txt Phylogenetic tree of NifH and NflH sequences and their accession numbers.

Supplemental Material mbio.01493-26-s0003.docx Figures S1-S5; Table S1 caption; Tables S2 and S3; caption for Files S1 and S2.

Table S1 mbio.01493-26-s0004.xlsx Plasmid names, organisms, DNA sequences, and protein accession numbers for the gene sets synthesized and expressed from plasmid pMTAP.

ASM does not own the copyrights to Supplemental Material that may be linked to, or accessed through, an article. The authors have granted ASM a non-exclusive, world-wide license to publish the Supplemental Material files. Please contact the corresponding author directly for reuse.
