# Supplementary material for: Functional methylthio-alkane reductases are widely distributed for bacterial organic sulfur acquisition
Source: mBio. 2026 Oct 6;17(11):e01493-26. doi: 10.1128/mbio.01493-26 (PMC13753814; doi:10.1128/mbio.01493-26)
Supplement: Supplementary file 1 [file mbio.01493-26-s0003.docx]

**Supplementary Information**

**Functional Methylthio-alkane Reductases are Widely Distributed for Bacterial Organic Sulfur Acquisition**

Ana Arroyo-Carriedo^1^, Elizabeth A. Morgan^1^, Adrienne B. Narrowe^2^, Rebecca A. Daly^2^, Emily Rogers^1^, Sarah J. Young^1^, Kelly S. Bender^3^, Kelly C. Wrighton^2^, Justin. A. North^1^*

1. Ohio State University, Department of Microbiology, Columbus, OH 43210

2. Colorado State University, Department of Crop and Soil Sciences, Fort Collins, CO

3. Southern Illinois University, School of Biological Sciences, Carbondale, IL

* Corresponding Author: [north.62@osu.edu](mailto:north.62@osu.edu); 614-292-4313

**Table of Contents:**

**Supplementary Figures S1-S5 and captions**

**Supplementary Tables S1-S3 and captions**

**Supplementary Files S1-S2 captions**

**Supplementary Figure S1. Dimethyl sulfide cleavage by Group IV-C clade NflD sequences.** The phylogenetic tree is a rectangular representation of the unrooted Group IV-C clade in Figure 1, with NflD accession numbers and corresponding organism. Coloredblocks represent the taxonomic class for each bacteria. Red and Black stars correspond to Group IV-C NflD sequences who are associated with a Group I and Group II NflH sequence, respectively, in the NFL gene cluster of the organism (see Supplementary Figure S3). Methane measurements of *R. rubrum* Δ*mar* Δ*nfl* strain complemented with NFL systems from Group IV-C grown in the presence of sulfate only (SO_4_^2-^; light gray) or sulfate plus dimethylsulfide (dark gray). Error bars are standard deviation for n=3 independent biological measurements. -- Dashed lines adjacent to methane measurement bars indicates those systems with DMS reduction activities that are statistically lower than the 10 µmol/L/O.D. cutoff for a physiologically relevant MAR system (P < 0.05, 1-tailed t-Test).

**Supplementary Figure S2. Ethyl methyl sulfide cleavage by Group IV-C clade NflD sequences.** The phylogenetic tree is a rectangular representation of the unrooted Group IV-C clade in Figure 1, with NflD accession numbers and corresponding organism. Colored blocks represent the taxonomic class for each bacteria. Red and Black stars correspond to Group IV-C NflD sequences who are associated with a Group I and Group II NflH sequence, respectively, in the NFL gene cluster of the organism (see Supplementary Figure S3). Ethane measurements of *R. rubrum* Δ*mar* Δ*nfl* strain complemented with NFL systems from Group IV-C grown in the presence of sulfate only (SO_4_^2-^; light gray) or sulfate plus ethylmethylsulfide (dark gray). Error bars are standard deviation for n=3 independent biological measurements. -- Dashed lines adjacent to ethane measurement bars indicates those systems with EMS reduction activities that are statistically lower than the 10 µmol/L/O.D. cutoff for a physiologically relevant MAR system (P < 0.05, one-sample 1-tailed t-Test).

**Supplementary Figure S3. Phylogenetic tree of NflH sequences with nitrogenase NifH and Isethionate Reductase IsrH sequences as the outgroups.** *Rhodospirillum rubrum* MarH sequence is highlighted for references, and NCIB accession numbers are provided as in Supplementary File S2.

**Supplementary Figure S4. Dimethyl sulfide cleavage is not observed in NFL systems from phylogenetic clades beyond Group IV-C.** Colored blocks represent the NFL clades as color coded in Figure 1. Red and Black stars correspond to Group IV NflD sequences who are associated with a Group I and Group II NflH sequence, respectively, in the NFL gene cluster of the organism (see Supplementary Figure S3). Methane measurements of *R. rubrum* Δ*mar* Δ*nfl* strain complemented with NFL systems from Group IV-A, IV-D, IV-E, and IV-F grown in the presence of sulfate only (SO_4_^2-^; light gray) or sulfate plus dimetylsulfide (dark gray). Error bars are standard deviation for n=3 independent biological measurements. -- Dashed lines adjacent to methane measurement bars indicates those systems with DMS reduction activities that are statistically lower than the 10 µmol/L/O.D. cutoff for a physiologically relevant MAR system (P < 0.01, one-sample 1-tailed t-Test).

**Supplementary Figure S5. Ethyl methyl sulfide cleavage is not observed in NFL systems from phylogenetic clades beyond Group IV-C.** Colored blocks represent the NFL clades as color coded in Figure 1. Red and Black stars correspond to Group IV NflD sequences who are associated with a Group I and Group II NflH sequence, respectively, in the NFL gene cluster of the organism (see Supplementary Figure S3). Ethane measurements of *R. rubrum* Δ*mar* Δ*nfl* strain complemented with NFL systems from Group IV-A, IV-D, IV-E, and IV-F grown in the presence of sulfate only (SO_4_^2-^; light gray) or sulfate plus ethylmetylsulfide (dark gray). Error bars are standard deviation for n=3 independent biological measurements. -- Dashed lines adjacent to ethane measurement bars indicates those systems with EMS reduction activities that are statistically lower than the 10 µmol/L/O.D. cutoff for a physiologically relevant MAR system (P < 0.01, 1-tailed t-Test).

**Supplementary Tables:**

**Supplementary Table S1: Excel File.** This file contains the plasmid names, organisms, DNA sequences, and protein accession numbers for the *nifX, nflB, nflH, nflD,* and *nflK* gene sets synthesized and expressed from plasmid pMTAP in this study.

**Supplementary Table S2: Control and modified plasmids used in this study**

| **Plasmid** | **Description** | **Use** | **Reference** |
| --- | --- | --- | --- |
| pMTAP-MCS3 | pBBR1-MCS3 backbone, pBBR Ori, *R. rubrum* MTA phosphorylase promoter, Tc^R^ | Broad host cloning vector for gene expression in *R. rubrum.* Serves as control vector when no NFL genes are inserted | Kovach 1995  Gene |
| pMTAP-B1C4 | pMTAP with *R. rubrum marBH* and *Clostridium sp.* DL-VIII *marDK* | Chimeric production of *R. rubrum* MarB and MarH with *C. sp.* MarD and MarK | This work |
| pMTAP-C5-marHDK | pMTAP with *Ruminiclostridium hungatei* *marHDK* only. | Production of only MarH, MarD, and MarK in *R. rubrum* | This work |
| pMTAP-H5-marHDK | pMTAP with *Propionispora sp. 2/2-37* *marHDK* only. | Production of only MarH, MarD, and MarK in *R. rubrum* | This work |

**Supplementary Table S3: Primers used in this study**

| **Plasmid** | **Primer name** | **Sequence, 5’-3’ (restriction site underlined)** | **Restriction enzyme** |
| --- | --- | --- | --- |
| pMTAP-H5-*marHDK* | H5_NdeI_F | ATATATATCATATGTCAAAGAAAATTAAGCAGATCGC | NdeI |
|  | H5_XhoI_R | ATATATATCTCGAGCAAAATATTTGGATTCTAAGCG | XhoI |
| pMTAP-C5-*marHDK* | C5_NdeI_F | ATATATATCATATGGCGAAGAAGATTAAACAAATCGCG | NdeI |
|  | C5_XhoI_R | ATATATATCTCGAGCAAAATATTTGGATTTTACGCCGAC | XhoI |

**Supplementary File S1.** Phylogenetic tree in Newick format of NifD and NflD sequences and their accession numbers from phylogenetic groups I, II, II, IV, and V.

**Supplementary File S2.** Phylogenetic tree in Newick format of NifH and NflH sequences and their accession numbers.
